# A Transcriptional Variant of Anaplastic Lymphoma Kinase Promotes Apoptosis in Ovarian High‐Grade Serous Carcinoma

**DOI:** 10.1002/mc.23928

**Published:** 2025-05-19

**Authors:** Ako Yokoi, Daigo Yoshimori, Yasuko Oguri, Miki Hashimura, Makoto Saegusa

**Affiliations:** ^1^ Department of Pathology Kitasato University School of Medicine Sagamihara Kanagawa Japan

**Keywords:** ALK^ATI^, apoptosis, high‐grade serous carcinoma, PARP1

## Abstract

The current study aims to delineate the role of a novel anaplastic lymphoma kinase (ALK) transcript, ALK^ATI^, in ovarian high‐grade serous carcinoma (HGSC). Overexpressed ALK^ATI^ exhibited both cytoplasmic and nuclear localization in HGSC cells, whereas full‐length ALK was predominantly cytoplasmic. ALK^ATI^ interacts with the DNA repair protein, poly (ADP ribose) polymer 1 (PARP1), and cells stably overexpressing ALK^ATI^ (OE‐ ALK^ATI^) were more sensitive to cisplatin‐induced apoptosis. Consistent with this, cleaved PARP1 levels were higher in HGSC tissue samples in areas with nuclear ALK immunoreactivity. The ratio of antiapoptotic BCL2 relative to proapoptotic BAX was significantly increased in OE‐ALK^ATI^ cells, despite the increase in apoptosis, suggesting that ALK^ATI^‐mediated apoptosis is independent of mitochondrion‐driven cell death. OE‐ALK^ATI^ decreased epithelial‐mesenchymal transition/cancer stem cell properties but did not alter proliferation rates, and nuclear ALK immunopositivity was not associated with clinicopathological factors or prognosis in HGSC. Together, our observations suggest that ALK^ATI^ sensitizes HGSC cells to apoptosis (probably though an association with PARP1) but this may have a relatively minor impact on tumor progression.

**Abbreviations**
ALK: anaplastic lymphoma kinase
ATI: alternative transcription initiation
HGSC: high‐grade serous carcinoma
PARP1: poly (ADP‐ribose) polymerase 1
ALDH1: aldehyde dehydrogenase 1
IHC: immunohistochemistry

## 1 Introduction

Ovarian carcinoma is the leading cause of mortality among all gynecological malignancies; this can be attributed to the presence of peritoneal metastases in 74% of cases at diagnosis [1, 2]. High‐grade serous carcinoma (HGSC) is by far the dominant subtype diagnosed clinically, and accounts for up to 80% of all ovarian cancer deaths [3, 4]. Delineating the molecular mechanisms involved in the initiation and progression of HGSC is therefore critical for the discovery of novel therapeutic agents to improve survival rates.

Anaplastic lymphoma kinase (ALK) is a 200‐kDa receptor tyrosine kinase encoded by the *ALK* gene on chromosome 2p23. The ALK protein consists of a large extracellular domain, a lipophilic transmembrane segment, and a cytoplasmic tyrosine kinase domain [5, 6]. Consistent with its role as an oncogenic driver, full‐length ALK and ALK translocations are found in primary solid tumors including neuroblastoma and lung carcinoma [7, 8].

An alternative transcription initiation (ATI) site located in intron 19 of the *ALK* gene leads to the expression of ALK isoform, ALK^ATI^, which encodes only the intracellular kinase domain [9, 10]. ALK^ATI^ is expressed in approximately 11% of melanoma cases, less frequency in other tumor types and is not present in normal tissues [9, 10].

We have shown that full‐length ALK is a critical driver of HGSC and engenders the aggressive phenotypic characteristics of HGSC [11]. Spurred by the discovery of ALK^ATI^ in other tumor types, we designed the current study to evaluate the role of this novel isoform in HGSC.

## 2 Materials and Methods

### 2.1 Plasmids and Cell Lines

ALK^ATI^ cDNA was amplified from a human full‐length ALK cDNA clone [11] by PCR using the specific primers, 5′‐ GCGAATTCATGCAGATGGAGCTGCAGAGCCC‐3′ and 5′‐ CGGGATCCGGGCCCAGGCTGGTTCATGC‐3′. The resulting PCR products, as well as full‐length ALK cDNA, were cloned into the p3xFLAG‐CMV‐14 vector (Sigma‐Aldrich Chemicals, St Louis, MO). Clones stably overexpressing ALK^ATI^ were established using the HGSC cell line, OVCAR‐3, as described previously [11].

### 2.2 Antibodies and Reagents

Antibodies are listed in Supplementary Table S1. Cisplatin (CDDP; P4394) and doxorubicin (Dox; D1515) were obtained from Sigma‐Aldrich Chemicals (St. Louis, MO, USA).

### 2.3 Western Blot and Co‐Immunoprecipitation (Co‐IP) Assays

Extraction of total cellular proteins, western blot, and co‐IP assays were carried out as described previously [11].

### 2.4 Flow Cytometry and Aldefluor Assay

Cell cycle analysis and ALDH1 enzyme activity using a fluorogenic dye‐based Aldefluor assay (Stem Cell Technologies, Grenoble, France) were performed as described previously [11].

### 2.5 Cell Counting Kit‐8 Assay

Cell viability after CDDP treatment was evaluated using the Cell Counting Kit‐8 (CCK‐8; Dojindo Lab, Kumamoto, Japan), according to the manufacturer's instructions.

### 2.6 Apoptotic Index

The number of apoptotic cells identified in HE‐stained sections was calculated by counting the mean number of apoptotic figures per field as described previously [12].

### 2.7 Immunofluorescence

After 10 μM CDDP treatment, cells were incubated with primary antibodies. Alexa 488 and 570 (Thermo Fisher Scientific, Waltham, MA, USA) were used as secondary antibodies. Cells that were double‐immunopositive for FLAG‐ALK^ATI^ and cleaved PARP1 were counted in five randomly selected high‐power fields (HPFs). The labeling indices (LIs) were then calculated per the number of FLAG‐ALK^ATI^ immunopositive cells.

### 2.8 Clinical Cases

We selected 150 cases of surgically resected HGSC (Supplementary Table S2) that were carried out between 2005 and 2019 at Kitasato University Hospital. Cases were chosen, according to the criteria of the 2014 World Health Organization classification, the TNM, and the International Federation of Gynecology and Obstetrics (FIGO) classification [13, 14, 15]. Thirty‐three cases received neoadjuvant paclitaxel/carboplatin‐based chemotherapy (NAC) before surgical treatment (Supplementary Table S2). NAC‐treated HGSC cases were subdivided into three groups on the basis of chemotherapy response score (CRS) for HGSC [16], as follows: CRS 1, no or minimal tumor response; CRS 2, partial response; CRS3, total or near‐total response. All tissues were routinely fixed in 10% formalin and processed for embedding in paraffin wax. Approval for this study was given by the Ethics Committee of the Kitasato University School of Medicine (B18‐048).

### 2.9 Immunohistochemistry (IHC)

IHC was performed using a combination of the microwave oven heating and polymer immunocomplex (Envision, Dako) methods as described previously [11, 12]. For evaluation of IHC findings, scoring of nuclear, cytoplasmic, or membranous immunoreactivities was performed [11, 12]. Briefly, the proportion of immunopositive cells among the total number of counted cells was subdivided into five categories as follows: 0, all negative; 1, < 10%; 2, 10–30%; 3, 30–50%; and 4, > 50% positive cells. The immunointensity was also subclassificed into four groups: 0, negative; 1, weak; 2, moderate; and 3, strong. IHC scores were generated by multiplication of the values of the two parameters. We subdivided the nuclear ALK scores into high and low categories by cutoff value (= 3, as well as 1, 2, 4, 6, and 8) on the basis of the mean value (= 2.3) (Supplementary Figure S1). Cleaved PARP1‐immunopositive cells were also counted in five HPFs that were randomly selected from nuclear ALK‐high and ALK‐low areas, respectively. We then calculated cleaved PARP1 LIs in these regions. In addition, p53 immunoreactivity was subdivided into mutant (mt, score 0 and 6‐12) and wild‐type (wt, score 1‐5) classes [17, 18].

### 2.10 Statistics

Comparative data were analyzed using the Mann–Whitney *U*‐test and Chi‐square test. Overall survival (OS) and progression‐free survival (PFS) were calculated as described previously [11]. The cutoff for statistical significance was set as *p* < 0.05.

## 3 Results

**Figure 1:**
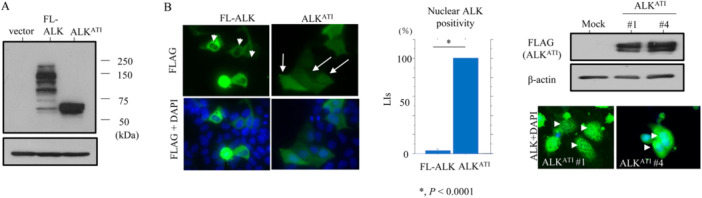
Both nuclear and cytoplasmic localization of ALK^ATI^ in HGSC cells. (A) Western blot analysis for the indicated proteins in total lysates from OVCAR‐3 cells transfected with empty vector, full‐length (FL) ALK, or ALK^ATI^. (B) Left: immunofluorescence of OVCAR‐3 cells transfected with full‐length (FL) ALK or ALK^ATI^. Note the lack of nuclear FL‐ALK staining (indicated by arrowheads), in contrast to distinct nuclear ALK^ATI^ staining (indicated by arrows). FL‐ALK and ALK^ATI^ were detected using anti‐FLAG antibody. Middle: the LIs of nuclear immunopositivity for FL‐ALK and ALK^ATI^ are shown as mean ± SD. Upper right: western blot analysis for the indicated proteins in total cell lysates from OE‐ALK^ATI^ and mock cells. Lower right: immunofluorescence of OE‐ALK^ATI^ cells. Note the distinct nuclear ALK^ATI^ staining (indicated by arrow‐heads).

**Figure 2:**
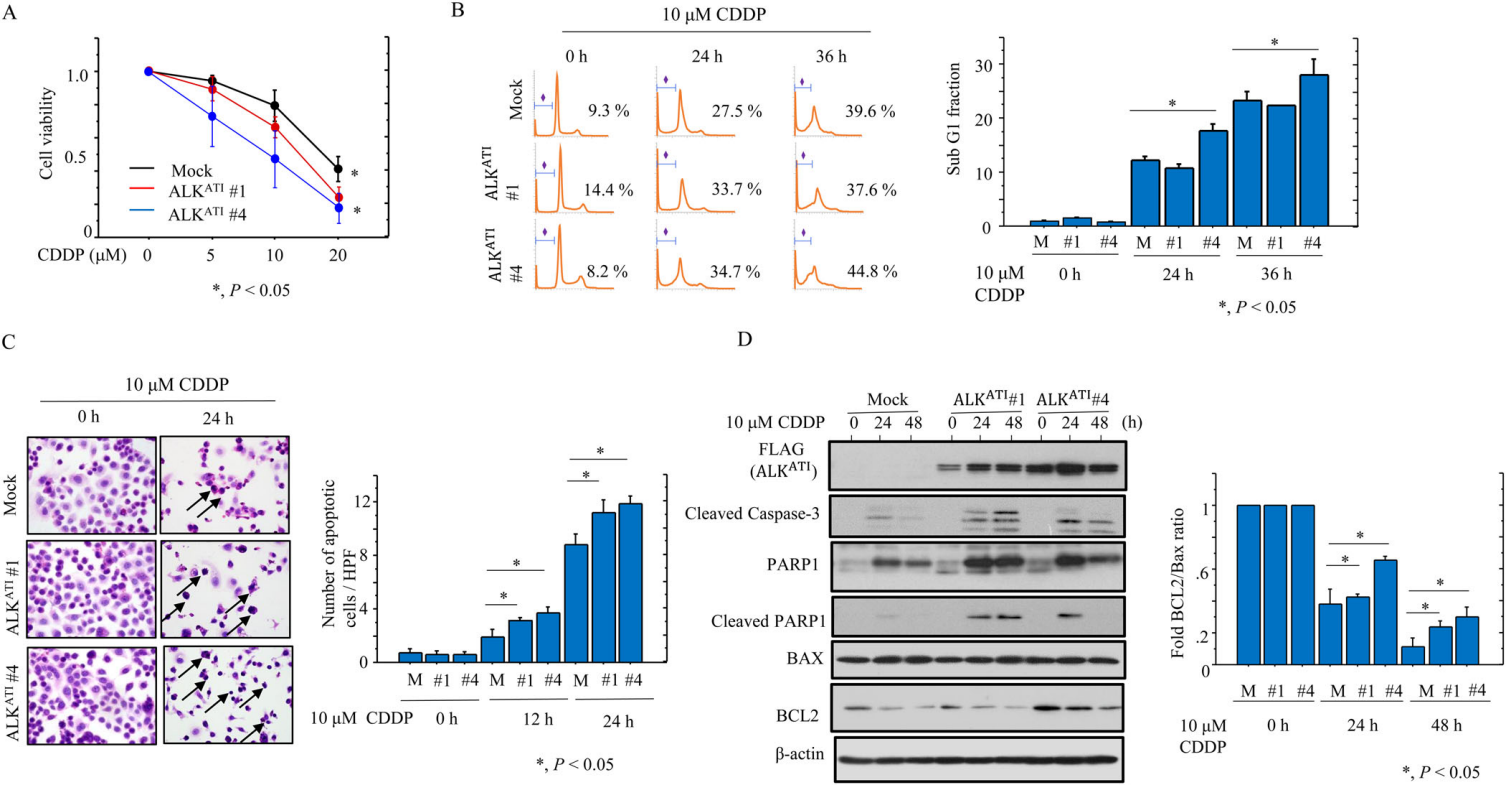
Changes in apoptosis following ALK^ATI^ overexpression in HGSC cells. (A) Treatment of OE‐ALK^ATI^ and mock cells with 10 μM CDDP for 24 h. Cell viability was measured using the CCK‐8 Viability kit. Viability for untreated cells is set as 1. (B) Left: flow cytometric cell cycle analysis for OE‐ALK^ATI^ and mock cells (M) after 10 μM CDDP treatment for the time shown. Daggers indicate sub‐G1 fractions. Right: the percentage of cells undergoing apoptosis (sub‐G1) was calculated and the data shown are as mean ± SD. The value in untreated cells is set as 1. (C) Left: arrows show OE‐ALK^ATI^ and mock cells (M) undergoing apoptosis after 10 μM CDDP treatment. Original magnification, x400. Right: numbers of apoptotic cells are shown as mean ± SD. (D) Left: western blot analysis of the indicated proteins in total lysates from OE‐ALK^ATI^ and mock cells with 10 μM CDDP treatment for the time shown. Right: the BCL2: BAX ratio was calculated following normalization to β‐actin using ImageJ version 1.41 (NIH, Bethesda, MD; http//imagej.gov/ij) and the data are shown as mean ± SD. Expression levels in untreated cells (0 h) were set as 1.

**Figure 3:**
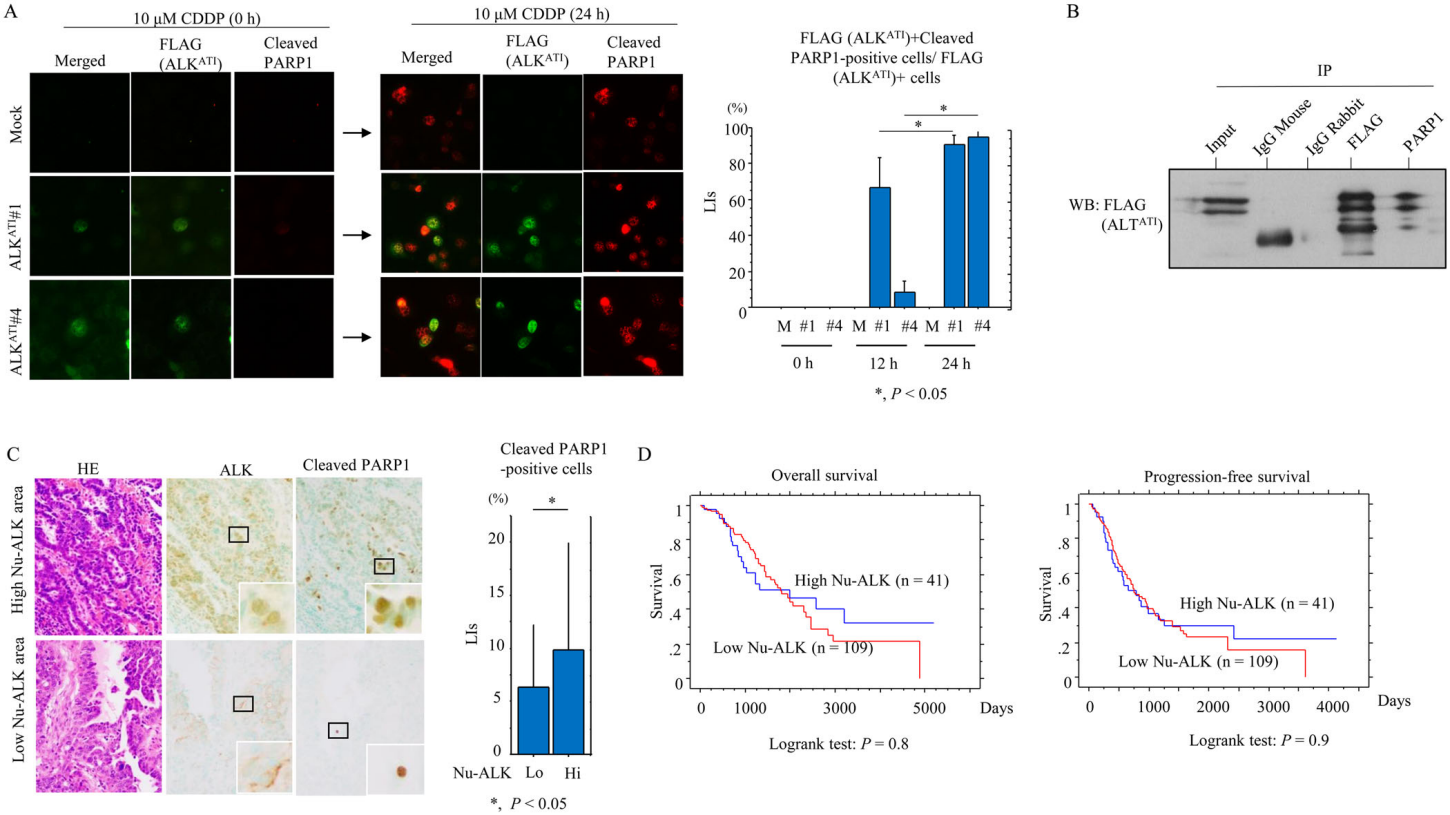
Relationship between ALK^ATI^ and PARP1 in HGSC**.** (A) Left: immunofluorescence of ALK^ATI^ and cleaved PARP1 in OE‐ALK^ATI^ and mock cells treated with 10 μM CDDP for the indicated times. Note the co‐immunolocalization of ALK^ATI^ and cleaved PARP1 in nuclei of cells treated with CDDP. Immunoreactivity for ALK^ATI^ is detected using anti‐FLAG antibody. Right: percentages of ALK^ATI^‐positive cells that are also positive for cleaved PARP1 are shown as mean ± SD. (B) After immunoprecipitation (IP) with the indicated antibodies using OE‐ALK^ATI^ cell lysates, we performed western blot (WB) using anti‐FLAG antibody. Input was 5% of the total cell extract. Normal rabbit and mouse IgGs were used as a negative control. (C) Left: staining with HE and IHC for the indicated proteins in samples with high and low nuclear (Nu)‐ALK^ATI^‐immunopositive (upper and lower panels, respectively). The closed boxes are magnified in the insets. Original magnification, x200 and x400 (insets). Right: cleaved PARP1 LIs in the high and low (Hi and Lo) Nu‐ALK^ATI^ ‐immunopositive categories. The LIs shown are mean ± SD. (D) OS (right) and PFS (left) relative to nuclear (Nu)‐ALK^ATI^ (low *vs.* high expression) in HGSC. n, number of cases.

Transcripts from the ATI site in intron 19 of the *ALK* gene result in three isoforms that encode proteins with predicted molecular weights of 61.1, 60.8, and 58 kDa [9, 10]. Consistent with this, we observed a major band of 60 kDa following transfection of ALK^ATI^ into OVCAR‐3 cells, whereas full‐length ALK migrated at around 200 kDa (Figure 1A). ALK^ATI^ exhibited both nuclear and cytoplasmic localization both when transiently overexpressed and when stably overexpressed in OVCAR‐3 cells (OE‐ALK^ATI^). In contrast, full‐length ALK was predominantly cytoplasmic (Figure 1B). OE‐ALK^ATI^ cells were significantly more sensitive to CDDP‐induced apoptosis when compared to mock cells (Figure 2A). Consistent with this, we observed an increased sub‐G1 fraction (Figure 2B) and more apoptotic cells (Figure 2C), as well as increased expression of ALK^ATI^, cleaved caspase‐3, PARP1, cleavedPARP1, and a higher BCL2: BAX ratio (Figure 2D). Similar findings were also observed in the Dox‐treated OE‐ALK^ATI^ cells (Supplementary Figure S2A). Co‐immunolocalization of nuclear ALK^ATI^ and cleaved PARP1 was significantly higher in OE‐ALK^ATI^ cells at 24 h post‐CDDP treatment when compared to 12 h (Figure 3A). This was consistent with the increased interaction between ALK^ATI^ and PARP1 observed following co‐IP experiments (Figure 3B). Heterogenous high nuclear ALK immunoreactivity (IHC score ≧ 3) was observed in 40 (26.6%) of 150 clinical HGSC tissue samples (Figure 3C). Of 33 cases (CRS1, 13 cases; CRS2, 20; CRS3, 0) receiving NAC, nuclear ALK scores appeared higher in patients with CRS 2 as compared to those of CRC1, although the difference was not statistically significant, probably due to the small number of cases investigated (Supplementary Figure S2B). Moreover, cleaved PARP1 immunoreactivity was significantly higher in ALK‐high nuclear immunopositive lesions when compared to the ALK‐low regions (Figure 3C). In contrast, nuclear ALK scores did not correlate with clinicopathological factors, OS, or PFS in HGSC, even when subdivisions were further made on the basis of five cutoff values (Figure 3D and Supplementary Table S2).

Since data from the TCGA Research Network indicate that HGSC is characterized by *TP53* mutations in up to 96% of the cases [19] and *TP53* wt HGSC are rare (< 1%) [20], we further examined whether p53 abnormality affects nuclear ALK immunoreactivity in HGSC. Although mt and wt p53 were found in 47 (81%) and 11 (18.9%) of 58 HGSC cases investigated, respectively, there was no association between nuclear ALK scores and p53 status (Supplementary Figure S3); this may be due to extremely high p53 abnormality in HGSC, in contrast to the nuclear ALK immunopositivity.

**Figure 4:**
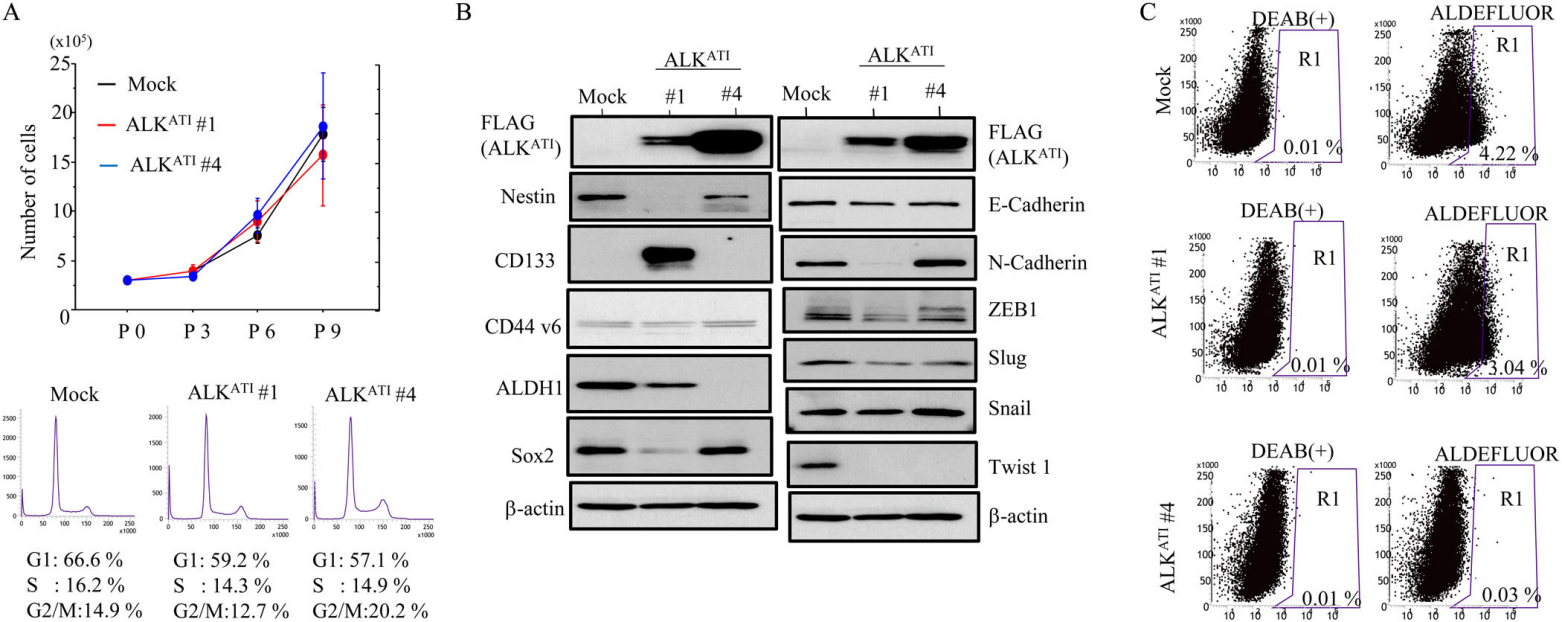
Relationships between ALK^ATI^, proliferation, and epithelial‐mesenchymal transition/cancer stem cell properties in HGSC cells. (A) Upper: OE‐ALK^ATI^ and mock cells were seeded at low density. P0, P3, P6, and P9 are 0, 3, 6, and 9 days after seeding, respectively. Lower: flow cytometry analysis of OE‐ALK^ATI^ and mock cells 3 days after seeding (P3). (B) Western blot analysis for the indicated proteins in total lysates from OE‐ALK^ATI^ and mock cells. (C) The percentage of live single‐cell populations, as well as the values of R1 in Aldefluor relative to R1 in DEAB(+) (indicated by parentheses), contained in each gate in OE‐ALK^ATI^ and mock cells are shown.

OE‐ALK^ATI^ cells had similar proliferative rates and cell cycle profiles as mock cells (Figure 4A). In contrast, levels of several epithelial‐mesenchymal transition (EMT)‐ and cancer stem cell (CSC)‐related markers including Nestin, ALDH1, ZEB1, Slug, and Twist 1 were lower in OE‐ALK^ATI^ cells when compared to mock cells (Figure 4B). Consistent with a lower level of CSC‐like properties, the frequency of ALDH1^high^ cells was low in the OE‐ALK^ATI^ cell population compared to mock cells (Figure 4C). In contrast, there were no associations between nuclear ALK scores and expression of several CSC markers in HGSC (Supplementary Figure S4); this may be due to the heterogenous distribution of nuclear ALK‐immunopositive cells within the tumors.

## 4 Discussion and Conclusions

We found that overexpression of ALK^ATI^ sensitizes HGSC cells to CDDP and is associated with the response to CDDP in clinical HGSC samples. Based on the colocalization and physical interaction of ALK^ATI^ and PARP1, we speculate that ALK^ATI^ may perturb PARP1‐dependent DNA repair pathways that are activated by genotoxins such as CDDP [21]. This would be consistent with the known regulation of DNA damage response, gene expression, and nuclear action structure formation by other tyrosine kinases [22, 23]. Since the BCL2:BAX ratio actually increases during CDDP‐ and Dox‐induced apoptosis in OE‐ALK^ATI^ cells, we suggest that ALK^ATI^‐mediated apoptosis is independent of ‘classical’ mitochondrion‐driven cell death.

There are conflicting results regarding the functional role of ALK^ATI^ during tumor progression. For example, ALK^ATI^ reportedly stimulates multiple oncogenic signaling pathways, mediates growth‐factor‐independent cell proliferation and promotes tumorigenesis [9]. ALK^ATI^ also acts as a potential prognostic factor and therapeutic target in soft tissue sarcoma [24]. In contrast, others have found that ALK^ATI^ expression is insufficient for cellular transformation or growth, and does not predict single agent therapeutic activity in melanoma cells [25]. In our results, cells overexpressing ALK^ATI^ had a similar proliferative rate to mock cells, but did display reduced EMT/CSC properties, although OE‐full‐length ALK cells enhanced cancer stem cell (CSC) features, increased cell proliferation, and accelerated cell mobility [11]. Moreover, nuclear ALK‐immunopositivity correlated neither with clinicopathological factors nor with prognosis in HGSC. Together, these data lead us to conclude that while ALK^ATI^ expression may sensitize HGSC cells to genotoxin‐induced apoptosis, it may play a relatively minor overall role in tumor progression.

## Author Contributions

Ako Yokoi, RO, and Makoto Saegusa carried out the majority of the experiments, analyzed the data, and wrote the manuscript. They were helped by Yasuko Oguri and Miki Hashimura. All authors reviewed and approved the final manuscript.

## Ethics Statement

This study was approved by the Kitasato University Medical Ethics Committee (B20‐181).

## Conflicts of Interest

The authors declare no conflicts of interest.

## Supporting information

Sup Figure S1.

Sup Figure S2.

Sup Figure S3 v2.

Sup Figure S4.

Supplementary Table S1.

Supplementary Table S2.

## Data Availability

The data that support the findings of this study are available on request from the corresponding author.
