# Supplementary figures and images for: A Transcriptional Variant of Anaplastic Lymphoma Kinase Promotes Apoptosis in Ovarian High‐Grade Serous Carcinoma
Source: Mol Carcinog. 2025 May 19;64(8):1281–6. doi: 10.1002/mc.23928 (PMC12272811; doi:10.1002/mc.23928)

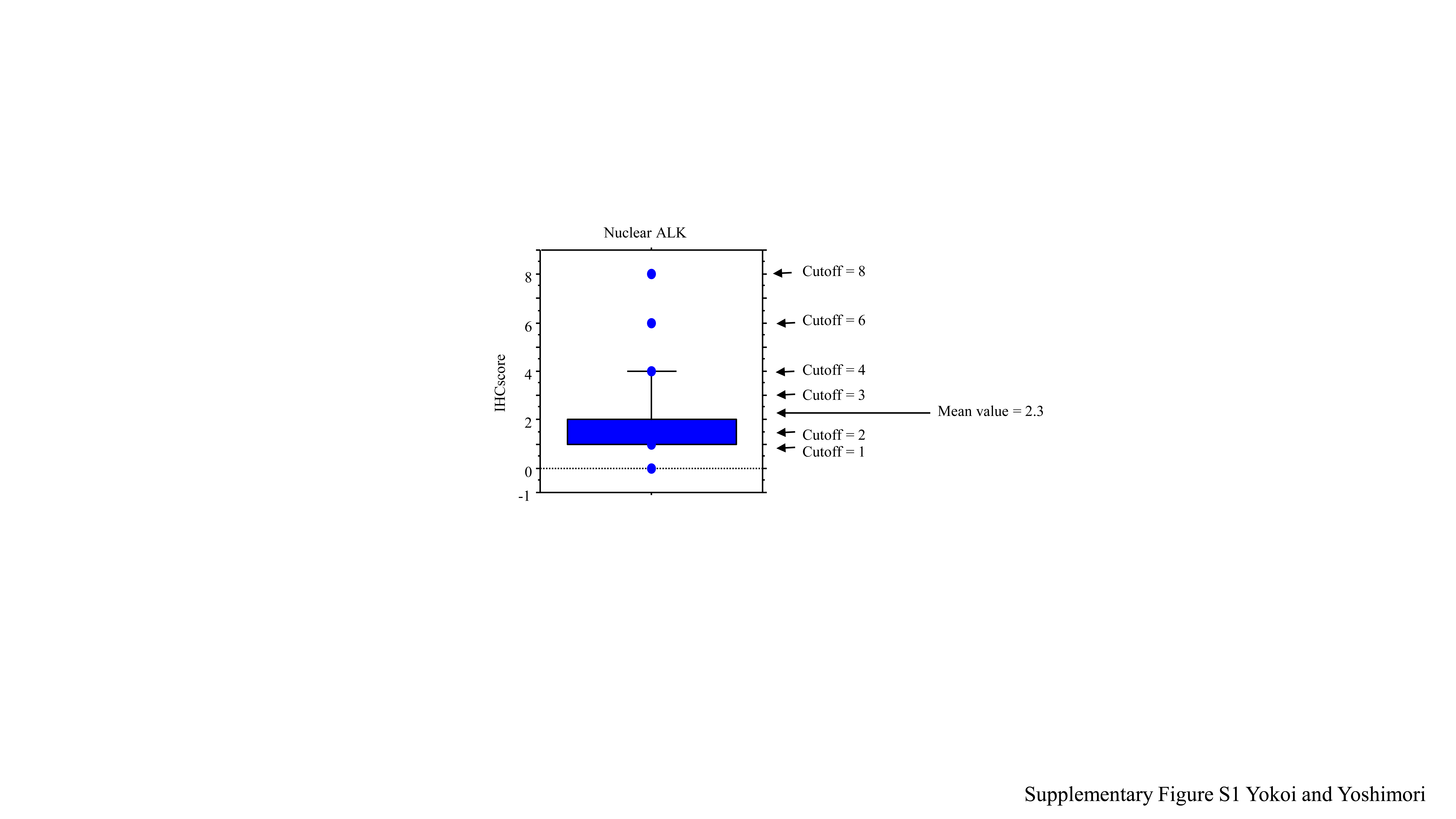

Supplement: Supplementary file 1 — Sup Figure S1. [file MC-64-1281-s006.tif]

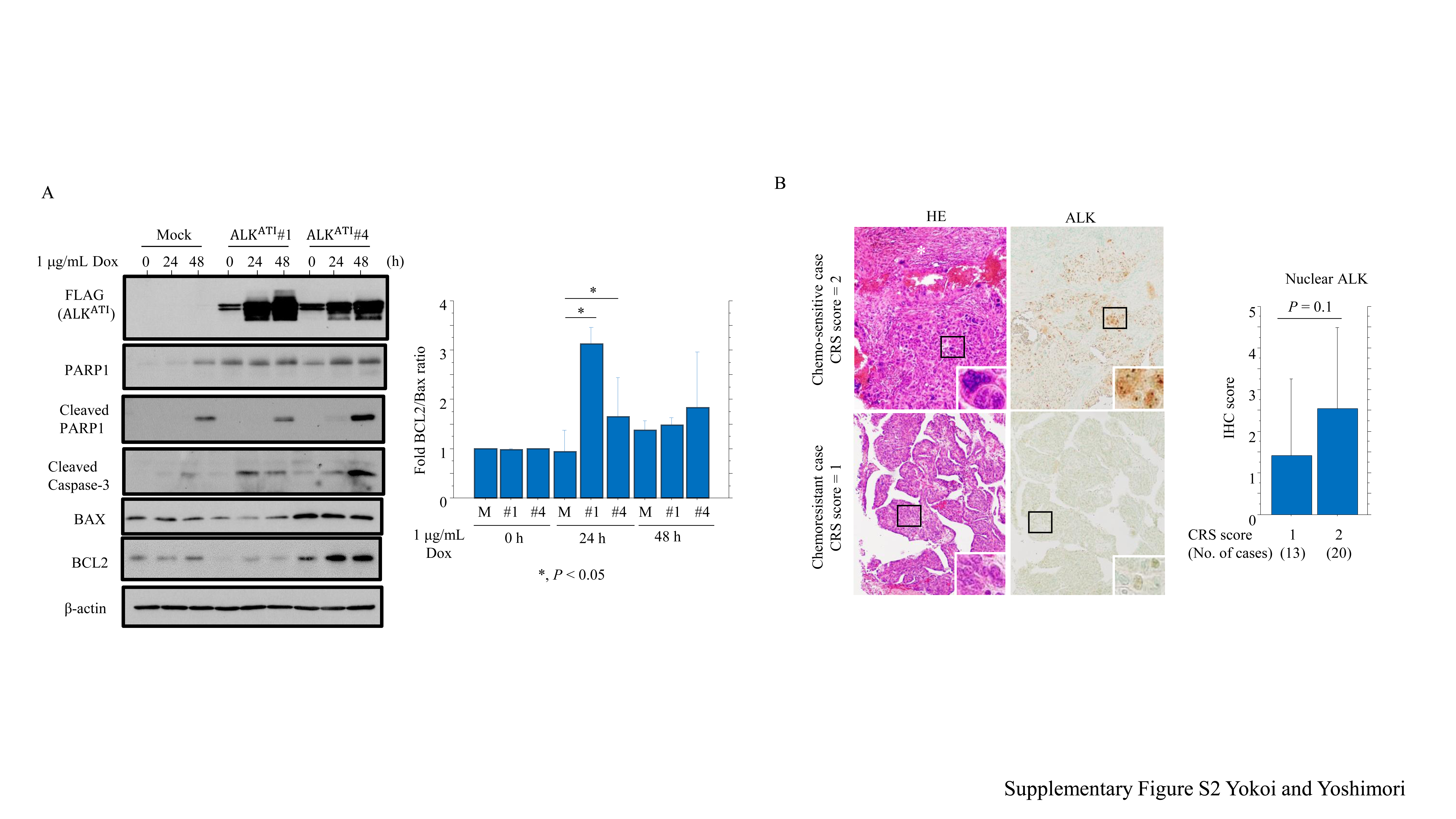

Supplement: Supplementary file 2 — Sup Figure S2. [file MC-64-1281-s001.tif]

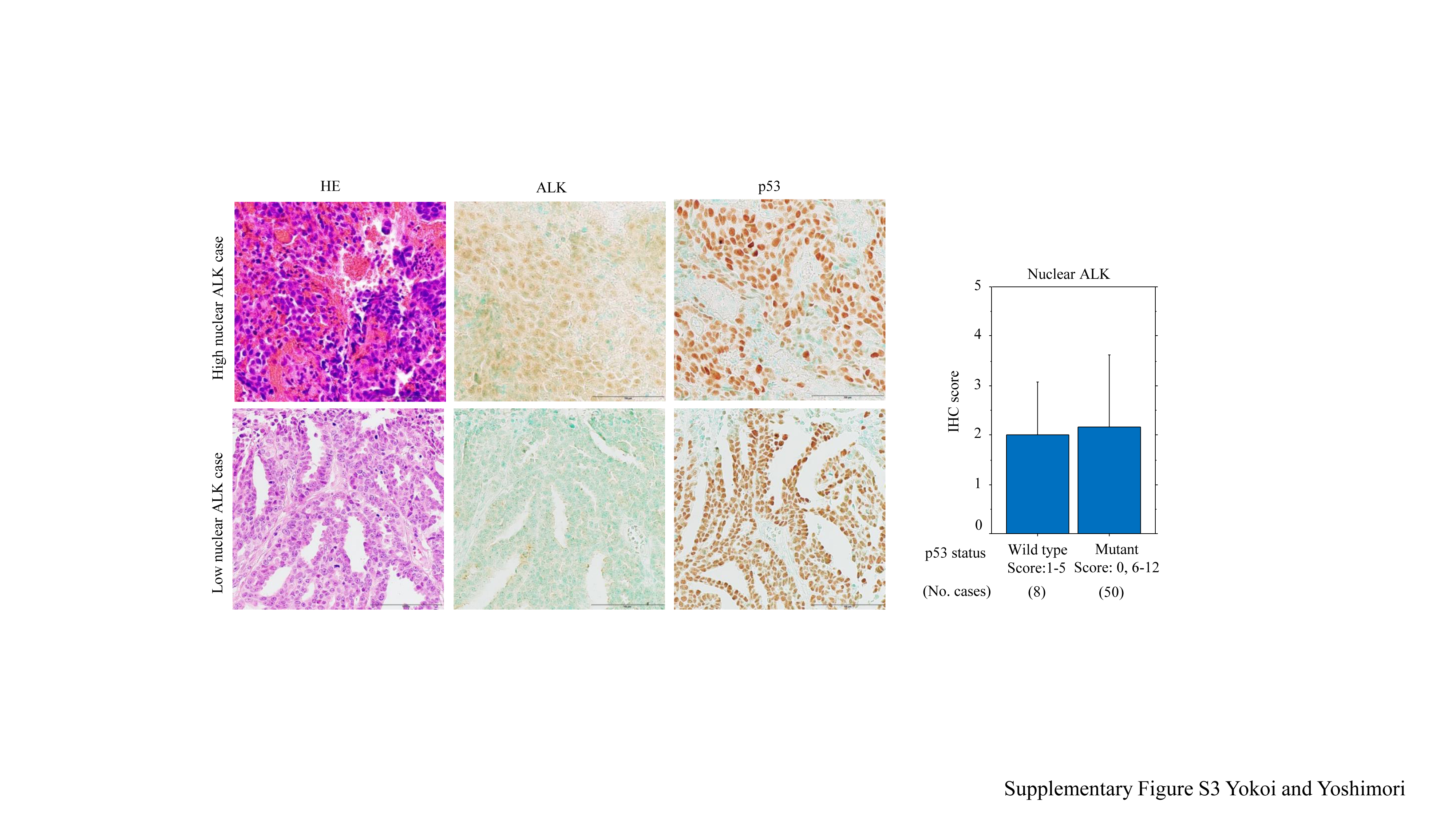

Supplement: Supplementary file 3 — Sup Figure S3 v2. [file MC-64-1281-s003.tif]

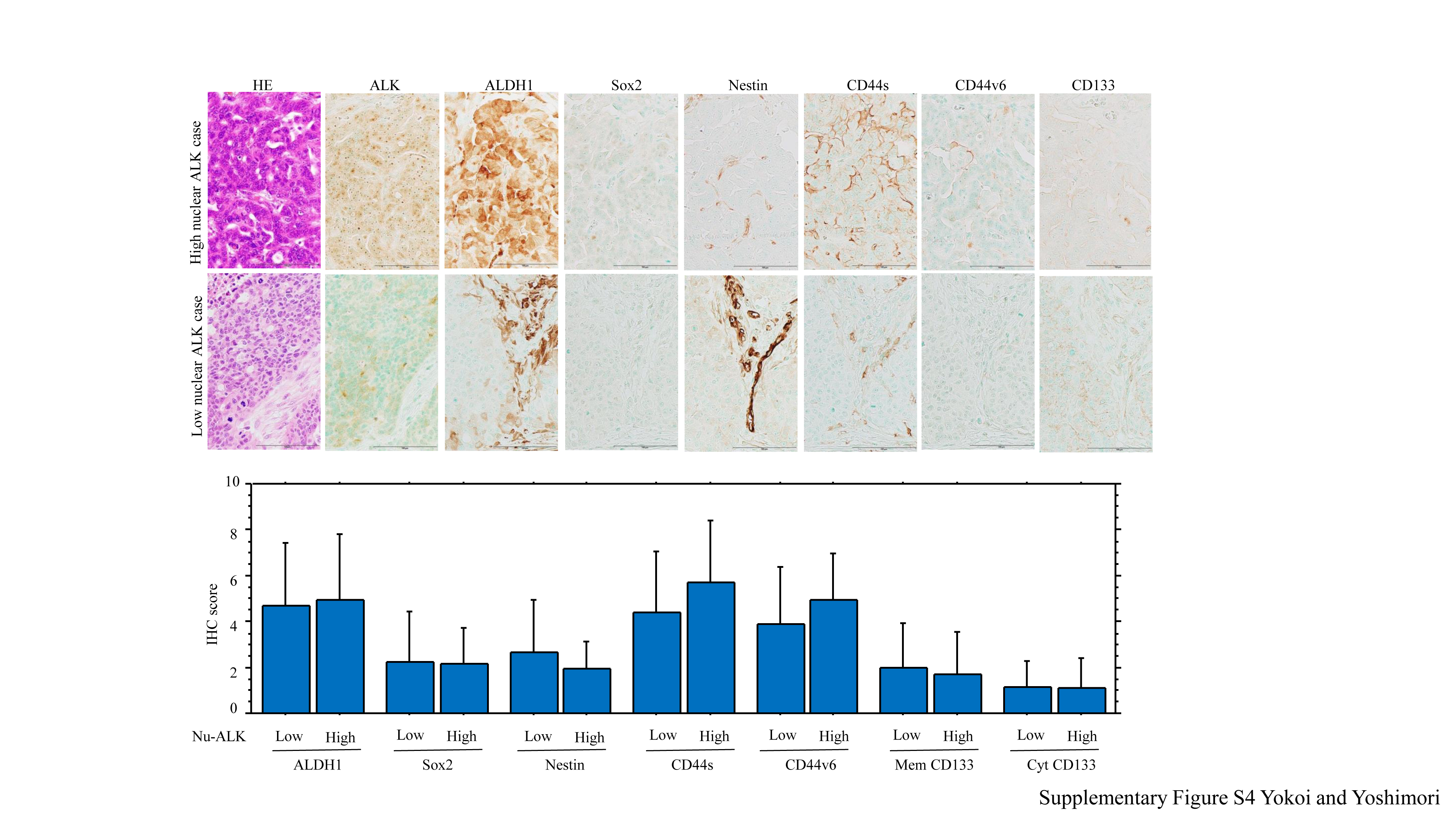

Supplement: Supplementary file 4 — Sup Figure S4. [file MC-64-1281-s002.tif]
